# World hepatitis day. Fighting hepatitis C in Latin America and the Caribbean; an urgent call

**DOI:** 10.7448/IAS.20.1.22183

**Published:** 2017-07-28

**Authors:** Luis Enrique Soto-Ramirez

**Affiliations:** ^a^ Department of Infectious Diseases, Instituto Nacional de Ciencias Médicas y Nutrición Salvador Zubirán, Mexico City, MEXICO

Chronic infection with hepatitis C virus (HCV) is a major and growing public health concern worldwide, with an estimate of 170 million individuals affected, 3% of the world´s population [1,2]. With more efficacious therapies becoming available, decision-makers will require accurate estimates of disease prevalence to assess the potential impact of new treatments, especially in places where prevalence is not well known. Few estimates of the epidemiologic burden are available for the Latin America and the Caribbean (LAC) region; and as a consequence, the potential impact of currently available treatments on the epidemiologic burden of HCV has not been assessed completely.

In the LAC region, most available data are local and national, rather than regional, and there are some gaps in terms of obtaining more accurate data and effectively preventing and controlling the disease. WHO’s framework for global action and Pan American Health Organization's (PAHO) regional strategy have helped in resolving these issues. There are several estimations on the number of HCV-infected individuals in LAC, some low 2.8–4.6 millions [3]; intermediate, 6.8–8.9 million [2]; and very high 9.8–11.9 millions [4]. Of those, only 25% have received the diagnosis, and 4% are being treated [5]. There are more than 350,000 deaths and 65,000 of persons per year that acquired the infection [6], but only one in four knows they are infected. Overall prevalence of anti-HCV antibodies is estimated to be 1.5% [2]. A meta-analysis made from LAC data found that injection drug users (IDU) presented the highest prevalence of HCV infection, from 1.7% in Colombia to over 95% in Northern Mexico. In non-injection drug users (NIDU), MSM and sex workers, prevalence is below 10% [7]. According to data from Argentina, Brazil, Mexico, Paraguay and Uruguay, 67% of the IDU population have anti-HCV antibodies; unfortunately this group has poor access to health services [2]. Other risk factors for HCV acquisition are also present in LAC: nosocomial infections, non-IDU injections, inadequate needle disposal, dental procedures and tattooing [1].

In a systematic review [5] presenting population-based estimates of HCV prevalence from general population and blood donor samples, of an estimated 7.8 million of HCV-chronically infected people in LAC, over 4.6 million would be expected to have genotype 1 (1a and 1b); and 1.6–2.3 million with genotype 1 would potentially benefit from current treatments. From those without treatment, one-third would be at risk of developing severe liver disease [8], contributing to an important cargo to health services in any country. These estimates demonstrate the substantial present epidemiologic weight of HCV and quantify the impending societal and clinical burden from untreated HCV in Latin America.

Between 2013 and 2014, the first direct acting antivirals (DAAs) were approved in the USA and the European Union. They are more effective, easier to administer, with minimal side effects, and had shown sustained virologic responses over 90% in 12- to 24-week regimens, in monoinfected and coinfected (with HIV) individuals [9]. Currently, LAC countries are planning their own strategies to use these drugs. However, the high cost of the new medications is the main factor that contributes to limit their accessibility; more cost-effective studies are needed to demonstrate this, as many decision-makers are not aware of the benefits. Moreover, we need a political commitment to include these drugs in the region, but unfortunately, corruption, lack of future planning and specially the absence of a public-health approach of many governments, as well as financial problems, are dramatically limiting the access to DAAs. As a real evidence of this situation, to this day, only 12/20 countries in LAC reported that hepatitis C test is free of charge, 5 more are free but only for specific groups; 12 states reported that at least one available drug for treating hepatitis C is on the national essential medicines list or subsidized by the government. However, drugs most commonly reported were pegylated interferon and ribavirin [10]. Seventeen countries that have signed agreements with PAHO, through the Strategic Fund, have received loans from PAHO for DAAs acquisition, so no excuse should exists for better treatments in the LAC region [11].This fund, created in 2000, provides DAAs at significantly lower prices as well as interest-free loans.

Different plans for DAAs acquisition have been established in the LAC region. Table 1 describes approaches and drug costs in four countries. While Brazil is already treating patients (36,000 in 2016), Argentina treated 1200 that year, whereas Mexico is now implementing treatment and Colombia started drug purchase. Brazil has negotiated lower prices due to a high volume purchases, but for smaller countries the PAHO Strategic Fund is their best option. Some countries like Brazil and Colombia have a history of seeking judicial remedies to access medicines, including ribavirine and alfa-peginterferon and, in selected, cases DAAs. This benefits only a small number of cases; however it is a good call for action [12].

**Table 1.** Current status of DAAs access in Argentina, Brazil, Colombia and México.

Country	HCV prevalence^[12]^ % (min-max) Number	PAHO strategic fund^[13]^	Access to DAAs[14]	Problems to solve	Sofosbuvir cost (USD/12 wks Rx)^†^
Argentina	1.51 (1.33–1.67) 609,541	Yes	Limited to government purchase.	Increase purchase of drugs and widening criteria to be a treatment candidate. Judicial initiatives to oppose drug patents	6258/1503^[15]*^
Brazil	1.64 (1.43–1.85) 3,299,281	Yes	To all candidates through the Ministry of Health.	Judicial initiatives to oppose drug patents	6875^[15]^
Colombia	3.14 (2.78–3.51) 1,462,378	Yes	Agreement with PAHO strategic fund 2017. ^[15]^	Implementation of treatment. ^[15]^	75,036.76 refunds/ 5408.96 OPS^[15]^
Mexico	1.35 (1.18–1.53) 1,601,583	No	Availability of DAAs through Mexican Social Security and Governmental Popular Insurance has been accepted and forthcoming implementation.	Increase purchase of drugs and widening criteria to be a treatment candidate. Create national centres to increase treatment access.	10,235.51

†Cost of DAAs for a 12-weeks treatment period in USD.

*Cost of generic drug.

World Hepatitis Day faces us to an unequal world, where amazing progress on anti-HCV treatments obtained during the last 25 years cannot be applied to all, leaving potentially curable patients to a natural history and progression to cirrhosis and hepatocarcinoma, creating large health costs for countries in the near future. We should be aiming at treating all affected, so please, we need that WHO and PAHO continue working in facilitating access to new drugs, that pharmaceutical companies offer the LAC region more affordable drug prices, and most of all, that health authorities and governments invest in their people´s future.

Since the first World Hepatitis Day in 2010, WHO, PAHO and many others have pushed for better and comprehensive diagnosis and care for individuals with any type of hepatitis. Despite all the efforts, as of today, the paramount challenges for the LAC region for hepatitis C still are to enhance preventing measures among high-risk populations, to increase awareness among the general population, to facilitate access to diagnosis and hopefully in the near future to augment the number of people treated and cured. It is urgent to stop hepatitis C, as it is curable and any human deserves a chance to be treated. HCV infection should be considered another important priority in the agenda for LAC countries [9].

## Abbreviations

DAAs, direct acting antivirals; HCV, hepatitis C virus;IDUs,injection drug users; LAC, Latin America and the Caribbean; NIDUs, non-injecting drug users.
